# Supplementary material for: Novel blood test for early biomarkers of preeclampsia and Alzheimer’s disease
Source: Sci Rep. 2021 Aug 5;11:15934. doi: 10.1038/s41598-021-95611-5 (PMC8342418; doi:10.1038/s41598-021-95611-5)
Supplement: Supplementary file 1 — Supplementary Information. [file 41598_2021_95611_MOESM1_ESM.pdf]

Novel blood test for early biomarkers of preeclampsia and Alzheimer's disease

Shibin Cheng[1]*, Sayani Banerjee[1]*, Lori A. Daiello[2], Akitoshi Nakashima[3], Sukanta Jash[1], Zheping Huang[1], Jonathan D. Drake[2], Jan Ernerudh[4], Goran Berg[4], James Padbury[1], Shigeru Saito[3], Brian R. Ott[2], and Surendra Sharma[1]**

[1]Department of Pediatrics, Women and Infants Hospital-Warren Alpert Medical School of Brown University, Providence, Rhode Island 02905, USA;

[2]Department of Neurology, Warren Alpert Medical School of Brown University and Alzheimer's Disease and Memory Disorders Center at Rhode Island Hospital, Providence, Rhode Island, 02903;

[3]Department of Obstetrics and Gynecology, University of Toyama, Toyama, Japan;

[4]Departments of Biomedical and Clinical Services, Linkoping University, Linkoping, Sweden

*S.C. and S.B. contributed equally to this work.

** Address correspondence to Surendra Sharma, MD, PhD, Department of Pediatrics, Women and Infants Hospital, 101 Dudley Street, Providence, Rhode Island 02905, USA. Phone: 401-430-8004.

Email: ssharma@wihri.org

Supplementary Figures:

Figure S1

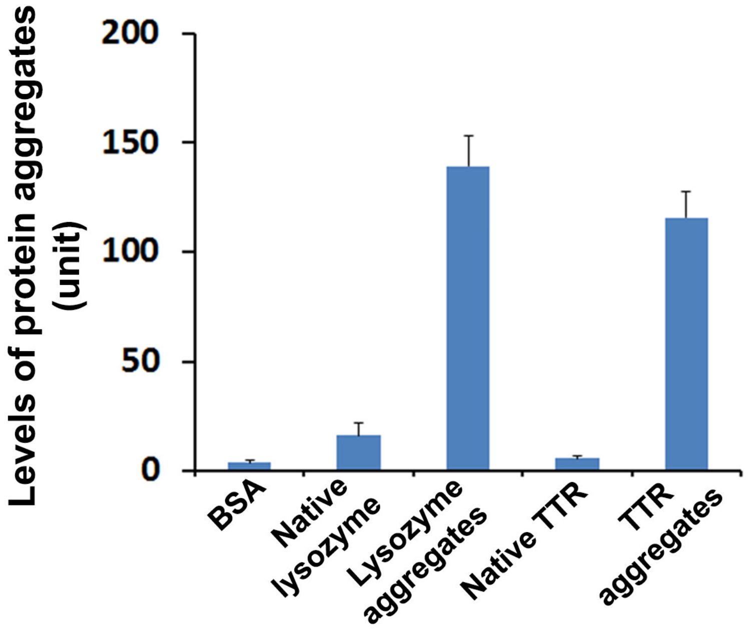

Figure S1. Validation of *in vitro* generated TTR aggregates. Strikingly higher levels of protein aggregates were detected in the solution containing our *in vitro* generated TTR aggregates and commercial lysozyme aggregates (Enzo) compared with BSA, native lysozyme and native TTR ($p < 0.001$). Protein aggregates were detected from indicated samples using ProteoStat protein aggregation assay kit.

Figure S2

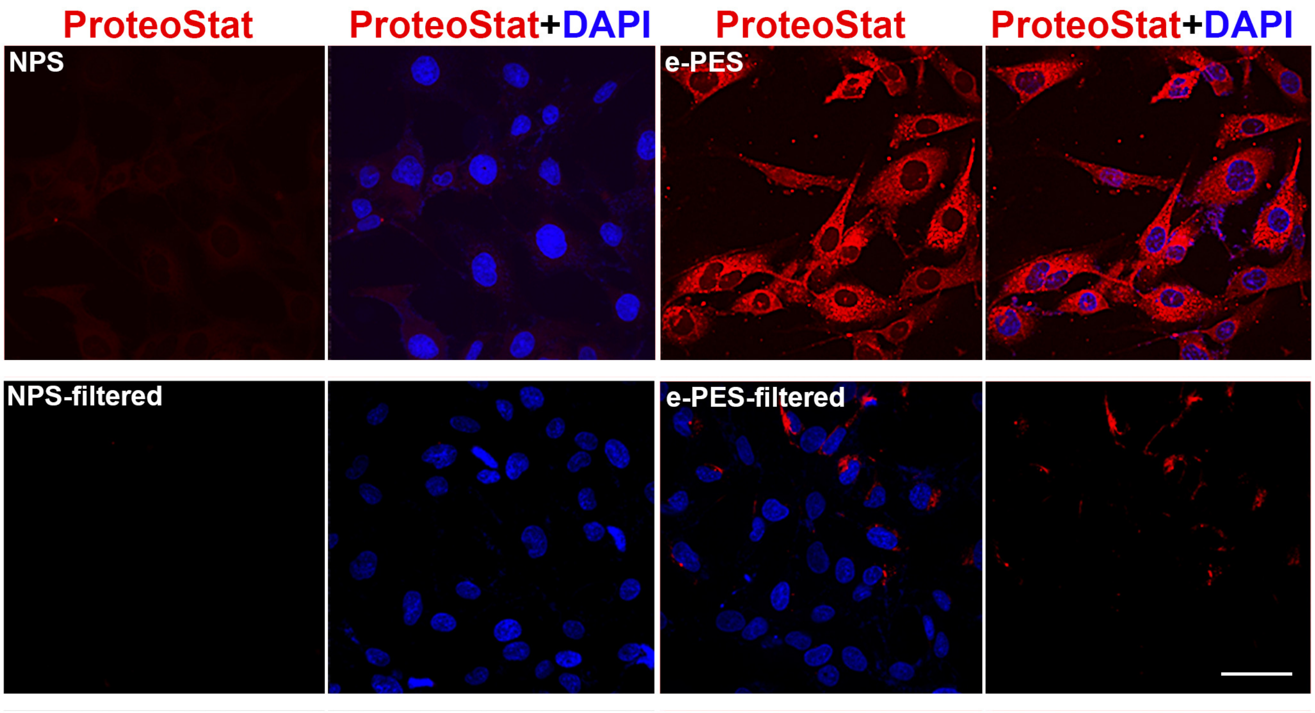

Figure S2. Depletion of the aggregates from e-PE sera attenuates the accumulation of protein aggregates in ADT. Cells were incubated for 24 h with e-PE sera or control sera before and after depletion of aggregates, fixed and then stained with ProteoStat dye. Images are representatives of at least 3 independent experiments. The nuclei were stained with DAPI (blue). Bar: 50 μm.

Figure S3

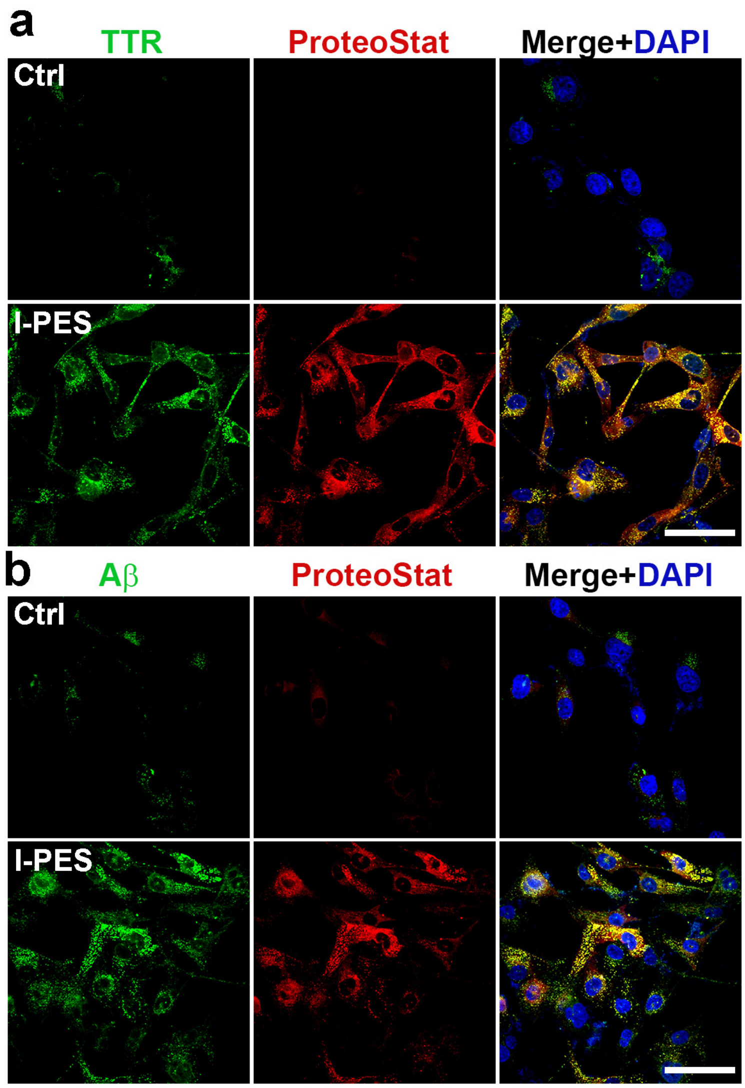

Figure S3. Identification of TTR and Aβ as components of the aggregates in ADT exposed to sera from l-PE (l-PES). ADT were incubated with l-PES or control sera (Ctrl), fixed at 24 h and immunostained for TTR (green, **A**) or Aβ (green, **B**) and then counter-stained with ProteoStat dye (red). The nuclei were stained with DAPI (blue). Images are representatives of at least 3 independent experiments. Bar: 50 µm.

Figure S4

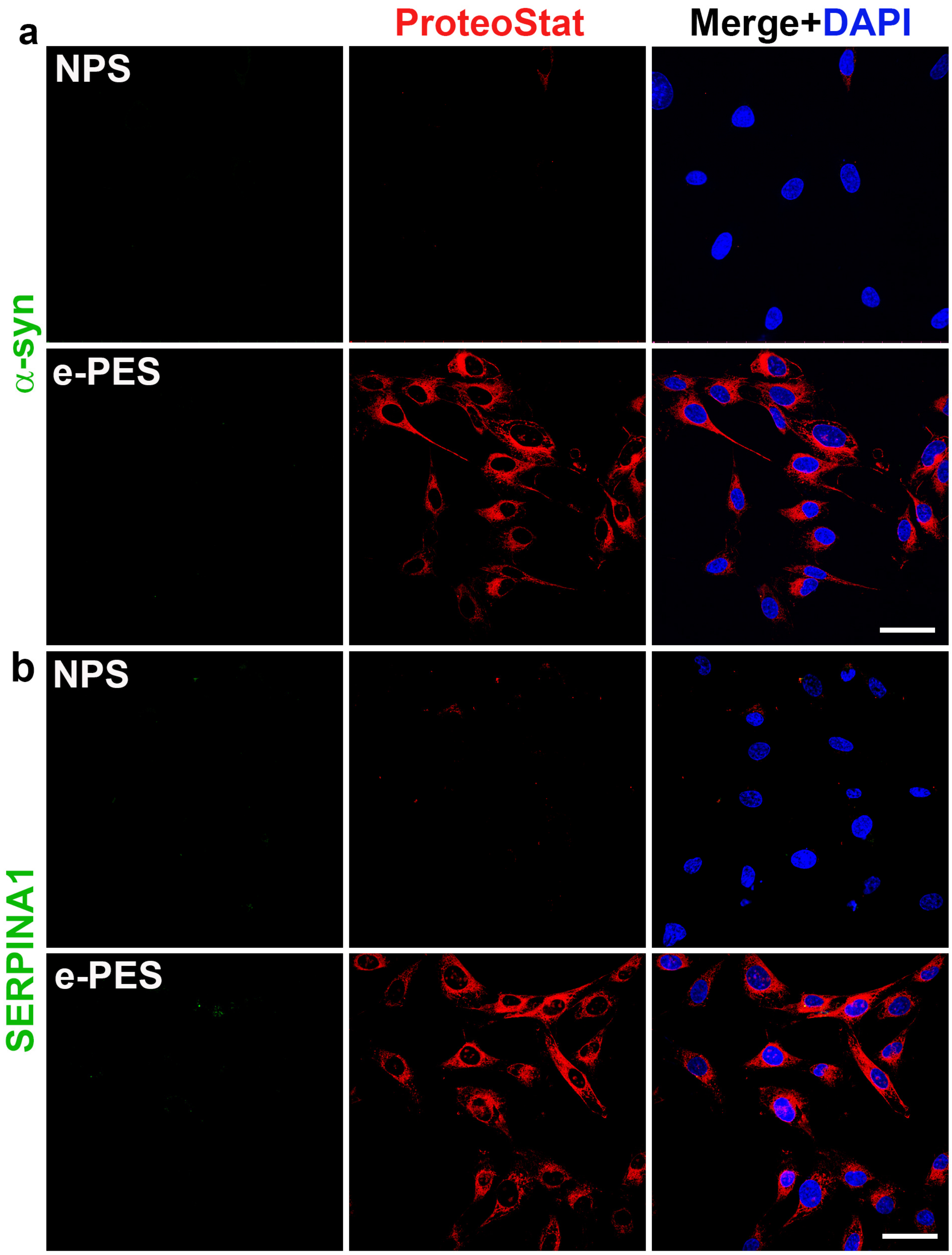

Figure S4. Aggregated α-synuclein and SERPINA1 are not detected in e-PE serum-treated ADT. ADT were incubated with e-PE sera (e-PES) or control sera (NPS), fixed at 24 h, immunostained for α-synuclein (α-syn, **A**) or SERPINA1 (green, **B**) and co-stained with ProteoStat dye (red). The nuclei were stained with DAPI (blue). Images are representatives of at least 3 independent experiments. Bar: 20 μm.

Table S1. Demographic data of patients with early-/late-onset preeclampsia and control pregnancies

Variable	l-PES (n=33)	NPS-l (n=38)	e-PES (n=33)	NPS-e (n=39)	*p*-value
Age (years)	29.4 (4.8)	29.9 (4.9)	30.9 (5.4)	29.6 (4.4)	NS
Gestational age at delivery (weeks)	37.7 (1.8)	37.5 (1.2)	30.2 (2.5)	29.9 (2.8)	NS
Maximum systolic blood pressure (mmHg)	>160	<120	>160	<120	†<0.01<br>††<0.01
Maximum diastolic blood pressure (mmHg)	>110	<80	>110	<80	†<0.01<br>††<0.01
Urine protein : creatinine	3.26 (3.11)	< 0.3*	5.0 (6.6)	< 0.3*	NA

l-PES: sera from late-onset PE

NPS-l: sera from normal pregnancy used as control for l-PES

e-PES: sera from early-onset PE

NPS-e: sera from normal pregnancy used as control for e-PES

Data presented as Mean (standard deviation) for continuous variables.

Data presented as n (%) for categorical variables.

*Serum creatinine, and urine protein:creatinine not mentioned in Control subjects and presented as normal ranges.

NS: not significant

† Late onset preeclampsia with severe features versus term controls.

†† Early onset preeclampsia with severe features versus preterm controls.

Table S2. Demographic data of patients with MCI and AD and age-matched cognitively normal controls

	Cognitive Normals (Controls) n = 19	MCI (Possible AD) n = 14	AD Dementia (Probable AD) n = 10
Age, mean, yrs (SD)	70.6 (9.3)	70.1 (8.5)	69.8 (9.5)
Sex, F/M, n	13/6	8/6	6/4
Education, mean, yrs (SD)	NA[†]	14.5 (2.8)	14.3 (2.0)
MMSE, mean, score (SD)	NA	26.5 (2.2)	16.8 (7.4)
CDR, 0.5/1/>1, n	NA	12/0/0	0/5/2
AD Biomarkers*, Amyloid PET+/CSF+/FDG-PET +, n	NA	11/1/0	3/2/1

AD: Alzheimer's Disease; SD: standard deviation; MMSE: Mini-Mental State Exam, range 0-30; CDR: Clinical Dementia Rating Scale, range (0-3); PET: Positron Emission Tomography; FDG-PET: ^{18}F-fluordeoxyglucose positron emission tomography

* AD Biomarker Positive (+) indicates: a) amyloid plaque burden (PET brain imaging), b) cerebrospinal fluid concentrations of amyloid-beta ($A\beta_{1-42}$), total tau (T-tau), and phosphorylated tau ($P\text{-}tau_{181}$) within established reference ranges for MCI or AD dementia, or c) neuronal dysfunction measured by FDG-PET. Number of subjects (n) who underwent biomarker analysis in each category is reported.

[†] Not assessed (NA)
